# Effect of Amorphous Metallic Fibers on Strength and Drying Shrinkage of Mortars with Steel Slag Aggregate

**DOI:** 10.3390/ma14185403

**Published:** 2021-09-18

**Authors:** Ji-Hwan Kim, Sung-Ho Bae, Se-Jin Choi

**Affiliations:** Department of Architectural Engineering, Wonkwang University, 460 Iksan-daero, Iksan 54538, Korea; 3869kjh@naver.com (J.-H.K.); caos1344@naver.com (S.-H.B.)

**Keywords:** amorphous metallic fiber, steel slag aggregate, mortar, strength, drying shrinkage

## Abstract

Recently, with increasingly stringent environmental regulations and the depletion of natural aggregate resources, high-quality aggregates have become scarce. Therefore, significant efforts have been devoted by the construction industry to improve the quality of concrete and achieve sustainable development by utilizing industrial by-products and developing alternative aggregates. In this study, we use amorphous metallic fibers (AMFs) to enhance the performance of mortar with steel slag aggregate. Testing revealed that the 28-day compressive strength of the sample with steel slag aggregate and AMFs was in the range of 48.7–50.8 MPa, which was equivalent to or higher than that of the control sample (48.7 MPa). The AMFs had a remarkable effect on improving the tensile strength of the mortar regardless of the use of natural aggregates. With AMFs, the drying shrinkage reduction rate of the sample with 100% steel slag aggregate was relatively higher than that of the sample with 50% natural fine aggregate. Furthermore, the difference in the drying shrinkage with respect to the amount of AMFs was insignificant. The findings can contribute to sustainable development in the construction industry.

## 1. Introduction

With the increasing stringency of various environmental regulations and the depletion of natural aggregate resources, the availability of high-quality aggregates is on the decline. Consequently, the construction industry has invested significant efforts toward enhancing the quality of concrete and achieving sustainable development through the utilization of industrial by-products and development of alternative aggregates [1,2,3,4,5,6,7,8,9,10,11].

In South Korea, owing to the development of the steel industry, tens of millions of tons of steel slags are generated every year. Blast furnace slag, a representative steel slag, has been widely used as an admixture material or a substitute for aggregates in the concrete industry; however, ferronickel slag, a by-product of the nickel industry, has not been utilized as much [12,13,14,15].

Some previous studies have used ferronickel slag in concrete [15,16,17,18,19,20]. Cho et al. [18] used ferronickel slag as a binder, and reported the reduction of pores inside the cement composites along with a reduction in the heat of hydration. Sarker et al. [19] used ferronickel slag as a substitute for natural sand (NS), and reported an enhancement in the fluidity and compressive strength of mortar at a replacement rate of 50% or less. Lee et al. [20] reported that when 30% or more ferronickel slag is used as a fine aggregate, the compressive strength is improved.

Recently, research on cement composites reinforced with amorphous metallic fibers (AMFs) possessing excellent mechanical properties and corrosion resistance has been attracting significant attention [21,22,23,24,25]. AMFs are known to be effective in improving the mechanical properties of mortar and concrete [22,24] and reducing plastic shrinkage cracks in cement composites [25]. Most existing studies employing AMFs [21,22,23,24] have used natural aggregates, and studies on AMF-reinforced cement composites with steel slag aggregates are scarce. To our best knowledge, there has been no study that used a mixture of blast furnace slag aggregate and ferronickel slag aggregate.

Hence, in this study, we used such a mixture and AMFs to enhance the performance of mortar with the steel slag aggregate, with the aim of contributing to the aforementioned efforts in sustainable development. The fluidity, compressive strength, tensile strength, flexural strength, drying shrinkage, and carbonation resistance of the mortar according to its AMF content were evaluated using a mixture of natural, blast furnace slag, and ferronickel slag aggregates.

## 2. Materials and Methods

### 2.1. Materials

The cement used in this study was ordinary Portland cement (OPC) from Asia Co., Korea. The following aggregates were obtained in the form of fine aggregates from POSCO Company of Pohang, Korea: NS (specific gravity = 2.60 g/cm^3^; fineness modulus = 2.89), blast furnace sand (BS) (specific gravity = 2.81 g/cm^3^; fineness modulus = 2.37), and ferronickel slag sand (FS) (specific gravity = 3.05 g/cm^3^; fineness modulus = 3.51). AMFs with a specific gravity of 7.2, tensile strength of 1400 N/mm^2^, thickness of 24 µm, and length of 15 mm (Seva, France) were used.

**Figure 1:**
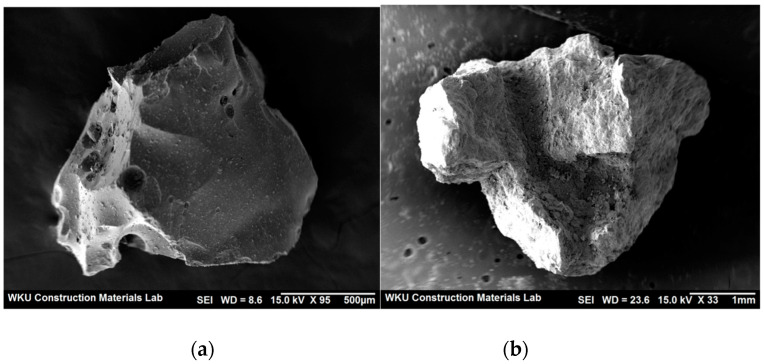
SEM images of steel slag fine aggregates: (**a**) BS and (**b**) FS.

**Figure 2:**
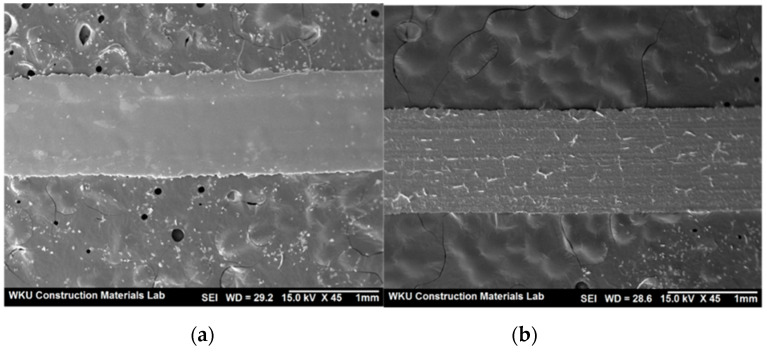
SEM images of amorphous metallic fibers (AMFs): (**a**) front side, (**b**) back side.

Figure 1 shows the SEM images of BS and FS. The surface of BS is smoother and contains more voids than that of FS. Figure 2 shows an SEM image of the AMFs, where one side has a smooth surface whereas the other side has a rough surface.

**Figure 3:**
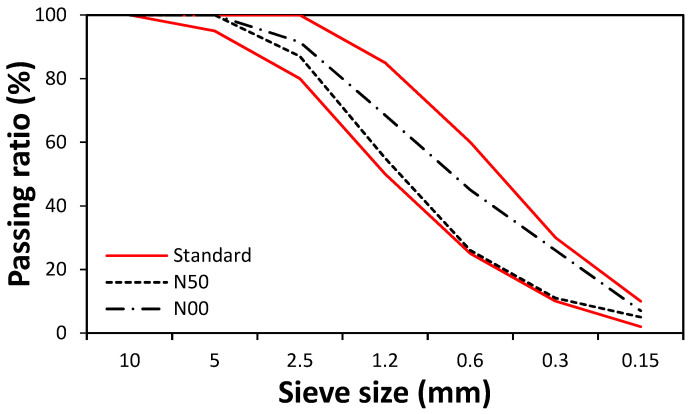
Particle size distribution of mixtures of fine aggregates.

The experiments were conducted using N50 mixtures containing 50% natural fine aggregate (NS 50%, BS 25%, and FS 25%) and N00 mixtures containing no natural fine aggregate (NS 0%, BS 50%, and FS 50%). Figure 3 shows that the passing ratios of the N50 and N00 mixed aggregates are within the standard range.

Table 1 summarizes the physical properties of the fine aggregates used in this study.

### 2.2. Mix Proportions and Specimen Preparation

Table 2 shows the mixture proportion of cement mortar. The water: cement ratio was fixed at 50%, and 0, 10, 20, and 30 kg/m^3^ of AMFs were used in the N50 and N00 mixtures. For comparison, a control sample was also prepared without the steel slag aggregate and AMFs.

Cubic specimens with an edge length of 50 mm were prepared for the compressive strength test, and cylindrical specimens with dimensions 50 mm × 100 mm were prepared for the split-tensile strength test. Furthermore, specimens with dimensions 40 mm × 40 mm × 160 mm were prepared for the flexural strength, drying shrinkage, and accelerated carbonation tests. The specimens were demolded after 24 h and cured in a water tank at 20 °C until the required age was attained.

The mortar flow and compressive strength were measured according to KS L 5105 [26]. For the compressive strength test, a load of 25 kN/s was applied. The tensile and flexural strengths were measured according to KS F 2423 [27] and KS F 2408 [28], respectively. The presented strength values are the average values of three samples.

The drying shrinkage was measured according to KS F 2424 [29] using a mechanical strain gauge. For the carbonation test, the carbonation depth was measured using a phenolphthalein solution after carbonation in an accelerated carbonation chamber at a constant temperature of 20 ± 2 °C, constant humidity of 60 ± 5%, and constant CO_2_ concentration of 5 ± 0.2%, according to KS F 2584 [30].

## 3. Results and Discussion

### 3.1. Mortar Flow

**Figure 4:**
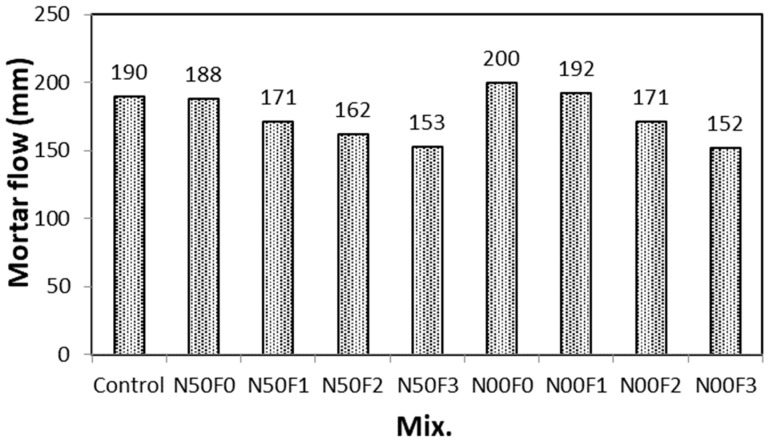
Mortar flow values for different aggregate mixtures.

**Figure 5:**
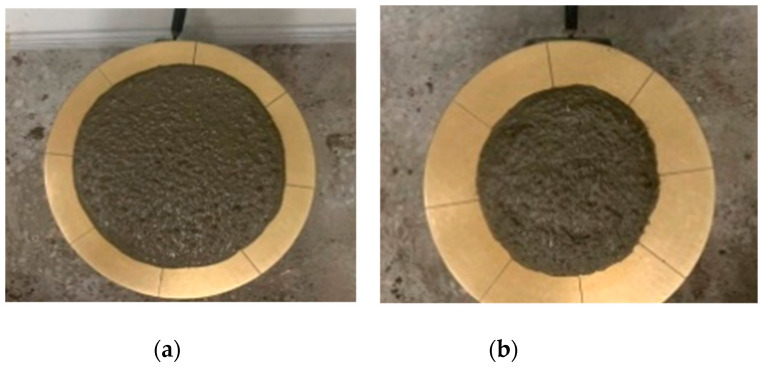
Mortar flow of (**a**) control (0 kg/m^3^ AMF) and (**b**) N00F3 (30 kg/m^3^ AMF) mixtures.

Figure 4 and Figure 5 show the change in flow with respect to the amount of AMFs in the mortar containing the steel slag aggregate. The flow of the control sample without the steel slag aggregate and AMFs is approximately 190 mm. As the amount of AMFs increases in the N50 and N00 mixtures, the mortar flow decreases. At the same amount of AMFs, the mortar flow of the N00 mixtures using only the steel slag aggregate is approximately 5.5–12.2% higher than that of the N50 mixtures using the 50% natural aggregate. This trend was also observed in the literature [19], where the workability of mortar using FS increased because the FS particles were coarser and the absorption rate was low.

However, for the N50F3 and N00F3 samples using 30 kg/m^3^ AMF, the mortar flow values are similar. This can be attributed to the increase in the amount of AMFs, as well as the dominant balling effect of the fibers compared to the influence of the characteristics of the steel slag aggregate. When a large amount of AMFs is used, the fiber balling effect may occur [31], and in this study, it appears that the mortar fluidity decreases due to the occurrence of this effect.

For the N50 mixtures, when the amount of AMFs is increased by 10 kg/m^3^, the mortar flow decreases by approximately 5.5–9.9%. Furthermore, for the N00 mixtures containing only the steel slag aggregate, a similar increase in the amount of AMFs decreases the mortar flow by approximately 4.1–13.2%.

### 3.2. Compressive Strength

**Figure 6:**
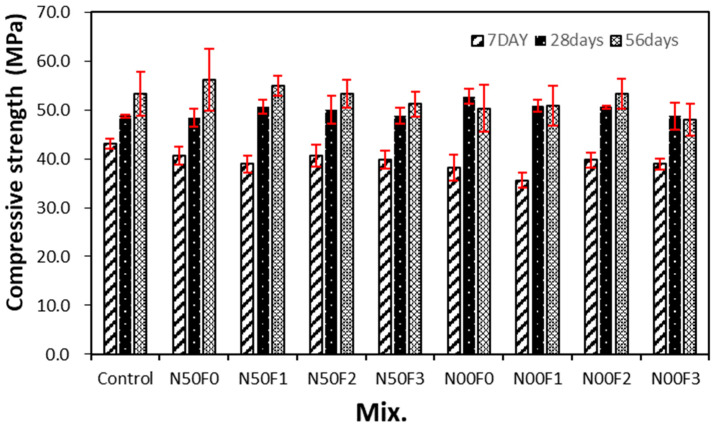
Compressive strength of different mixture samples.

Figure 6 shows the change in the compressive strength with respect to the amount of AMFs in the mortar containing the steel slag aggregate. For 7-day aging, the compressive strength of the control sample is the highest at approximately 43.0 MPa, while those of all samples using the steel slag aggregate are lower. The 7-day compressive strengths of the N50F2 and N00F2 mixtures using 20 kg/m^3^ AMF are higher than those of the other samples, regardless of the use of NS.

After 28 days of aging, the trend of compressive strength is different from that observed for 7 days of aging. The compressive strength of the sample containing the steel slag aggregate and AMFs is in the range of 48.7–50.8 MPa, which is equivalent to or higher than that of the control sample (48.7 MPa). In addition, the compressive strengths of the samples with 10 and 20 kg/m^3^ AMF are higher than those of the samples with 30 kg/m^3^ AMF.

At 56 days of aging, the compressive strength of the control sample is approximately 53.2 MPa, and that of the N50F0 sample using 50% of natural fine aggregate and no AMFs is approximately 56.1 MPa, which is approximately 5.4% higher than that of the control sample. With the same amount of AMFs, the 56-day compressive strength of the N50 mixtures is slightly higher than that of the N00 mixtures.

### 3.3. Tensile Strength

**Figure 7:**
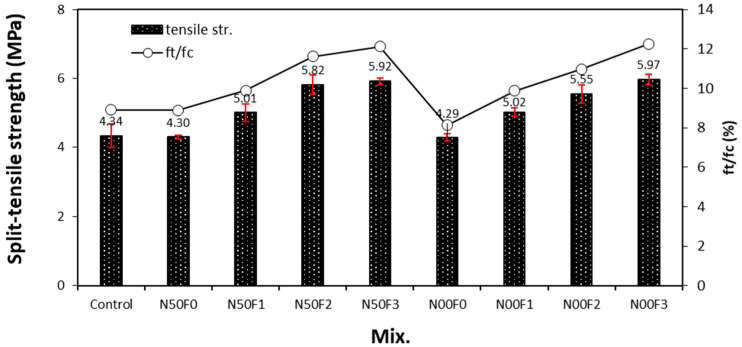
Tensile strengths of different mixture samples.

Figure 7 shows the change in the split-tensile strength with respect to the amount of AMFs in the mortar containing the steel slag aggregate. The 28-day tensile strength of the control sample is approximately 4.34 MPa, and those of the N50F0 and N00F0 samples without AMFs are approximately 4.29–4.30 MPa, similar to the tensile strength of the control sample. In addition, unlike the trend observed for the compressive strength, the tensile strength of all samples increases with increasing amount of AMFs.

The tensile strength of the N50 mixtures using 50% natural aggregate and AMFs is 5.01–5.92 MPa, which is 15.4–36.4% higher than that of the control sample. Even for N00 mixtures using only the steel slag aggregate, the tensile strength of the sample with AMFs is 5.02–5.97 MPa, which is 15.6–37.5% higher than that of the control sample.

Thus, AMFs have a significantly positive influence on the tensile strength of mortar containing steel slag aggregate regardless of the use of NS. Particularly, with an increase in the AMF content by 10 to 20 kg/m^3^, the tensile strength of the mortar increases by more than 10%; however, a further increase in the AMF content to 30 kg/m^3^ results in a reduced increase in the tensile strength, by 1.7–7.5%. Therefore, considering the tensile performance and economic feasibility of the mortar containing the steel slag aggregate, the optimum amount of AMFs that should be used is approximately 20 kg/m^3^. The tensile strength: compressive strength ratio of the sample without AMFs was approximately 8%–9%, whereas that of the samples with AMFs was higher, at approximately 10–12%.

### 3.4. Flexural Strength

**Figure 8:**
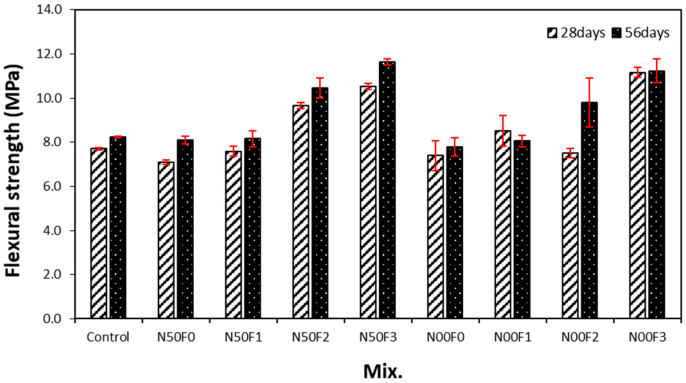
Flexural strengths of different mixture samples.

Figure 8 shows the change in flexural strength with respect to the amount of AMFs in the mortar containing the steel slag aggregate. Similar to the trend for the tensile strength, the flexural strength of the mortar increases with increasing amount of AMFs.

For 28 days of aging, the flexural strength of the mixtures using AMFs is 7.4–11.1 MPa, while for 56 days of aging, it is 8.10–11.6 MPa. The flexural strength increases with increasing age. Furthermore, the increase in flexural strength is higher for the sample using 20 kg/m^3^ or more AMFs than that for the sample using 10 kg/m^3^ AMF. In other words, for 56 days of aging, the flexural strength of the N50F1 and N00F1 samples using 10 kg/m^3^ AMF is approximately 8.1 MPa, which is approximately 0.6–3.8% higher than that of the sample without AMFs. On the other hand, the flexural strength of the N50F2 and N00F2 samples using 20 kg/m^3^ AMF is approximately 9.8–10.5 MPa, which is approximately 20.9–28.8% higher than that of the sample with 10 kg/m^3^ AMF. In addition, the 56-day flexural strength of the N50 samples using 50% natural aggregate is slightly higher than that of the N00 samples using 100% steel slag aggregate.

### 3.5. Drying Shrinkage

**Figure 9:**
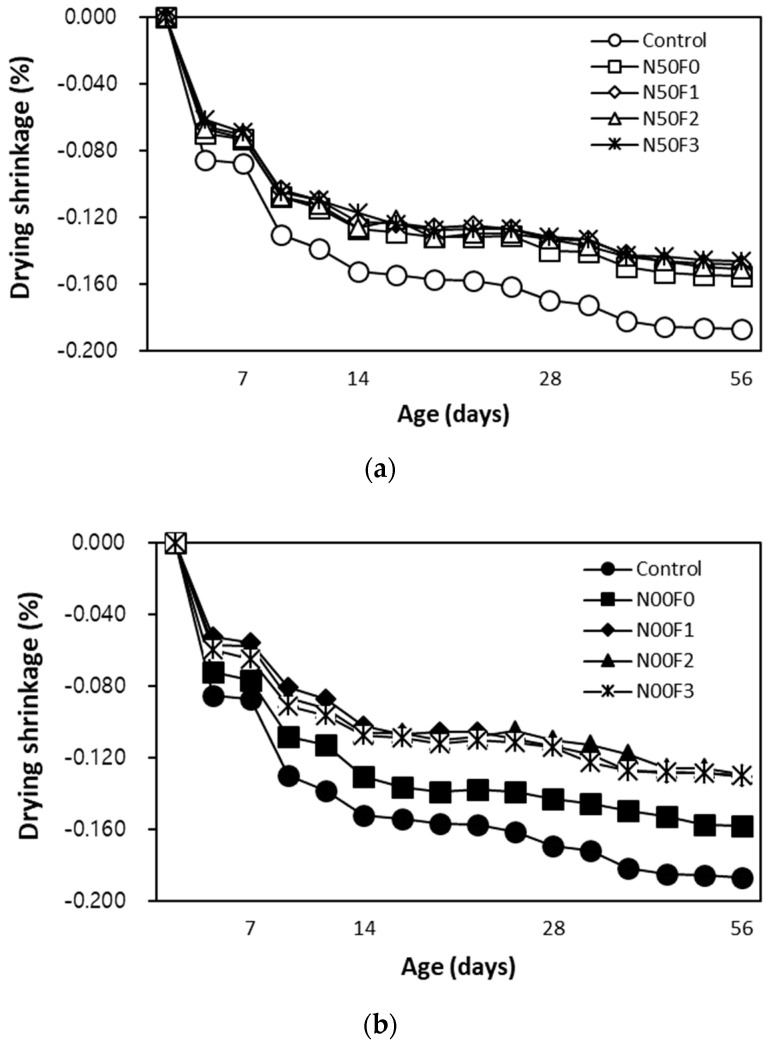
Drying shrinkage of (**a**) N50 and (**b**) N00 mixtures with 50% natural and 100% steel slag aggregates, respectively, as a function of age.

Figure 9 shows the changes in the drying shrinkage of mortar using steel slag aggregate and AMFs with respect to the age (in days). The drying shrinkage after 56 days of aging the control sample is the highest at approximately 0.186%. Figure 9a shows that the drying shrinkage of the N50F0 sample without AMFs is approximately 0.155%, while that of the sample with AMFs is approximately 0.145–0.150%, which is approximately 24.0–28.2% lower than that of the control sample.

For the N00 samples containing 100% steel slag aggregate (Figure 9b), the drying shrinkage of the N00F0 sample without AMFs is approximately 0.158%, while that of the sample with AMFs is approximately 0.130%, which is approximately 43% lower than that of the control sample.

In this study, when AMFs were used, the drying shrinkage reduction rate of the sample using 100% steel slag aggregate was relatively higher than that of the sample using 50% natural fine aggregate. Therefore, it is considered that the drying shrinkage of mortar and concrete can be effectively reduced when steel slag aggregate and AMFs are appropriately used. In addition, it was found that the change in the drying shrinkage with respect to the amount of AMFs used was insignificant.

### 3.6. Accelerated Carbonation Depth

**Figure 10:**
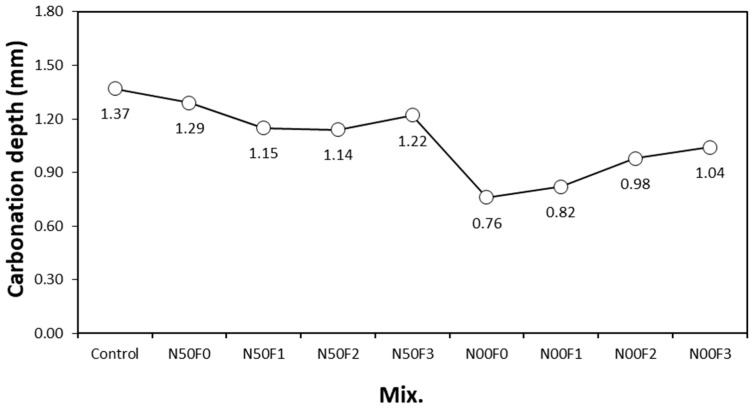
Carbonation depth of different mixture samples.

Figure 10 shows the change in the accelerated carbonation depth with respect to the amount of AMFs in the mortar containing the steel slag aggregate. The 28-day accelerated carbonation depth of the control sample is the largest at approximately 1.37 mm, whereas those of all samples using the steel slag aggregate and AMFs are lower.

In addition, the carbonation depths of the N00 samples, which used more steel slag aggregate, and those of the N50 samples, were lower than those of the N50 samples. For the N50 mixtures using 50% natural fine aggregate, the carbonation depth of the sample using AMFs was relatively low, and appeared to be similar regardless of the amount of AMF used. For the N00 mixtures using 100% steel slag aggregate, the carbonation depth of the mortar sample increased as the amount of AMF increased. This could be because the inflow of CO_2_ into the voids of the bond surface between the cement matrix and AMFs in the N00 samples was relatively higher than that in the sample without AMFs. However, the carbonation depth of the N00F3 sample, which was largest among the mortar samples containing 100% steel slag, was also lower than that of the control sample or those of the N50 samples.

## 4. Conclusions

The conclusions of this study can be summarized as follows.

- After 28 days of aging, the compressive strength of the sample using the steel slag aggregate and AMFs was 48.7–50.8 MPa, which was equivalent to or higher than that of the control sample (48.7 MPa). In addition, the compressive strengths of the samples with 10 and 20 kg/m^3^ AMF were approximately 3.6–4.3% higher than those of the samples with 30 kg/m^3^ AMF.
- As the amount of AMFs increased, the tensile strength of all the samples increased. AMFs had a notable effect on improving the tensile strength of mortar using the steel slag aggregate regardless of the use of natural aggregate.
- The increase in the flexural strength was larger in the sample using 20 kg/m^3^ or more of AMFs than that in the sample using 10 kg/m^3^ AMF.
- After 56 days of aging, the drying shrinkage of the control sample was the highest at approximately 0.186%. For the N00 samples using 100% steel slag aggregate, the drying shrinkage of the N00F0 sample without AMFs was approximately 0.158%. Furthermore, the drying shrinkage of the sample using AMFs and 100% steel slag aggregate was approximately 0.130%, nearly 43% lower than that of the control sample.
- The 28-day accelerated carbonation depth of the control sample was the largest at approximately 1.37 mm, whereas those of all samples using the steel slag aggregate and AMFs were approximately 11–40% lower.

Further studies are required to establish the relationship between the microstructures of cement composites and mechanical properties depending on the presence of AMFs and steel slag aggregate.

## Figures and Tables

**Table 1:** Physical properties of fine aggregates.

Type	Fineness Modulus (FM)	Density (g/cm^3^)	Water Absorption Ratio (%)
Natural sand (NS)	2.89	2.60	1.0
Blast furnace slag fine aggregate (BS)	2.37	2.81	2.1
Ferronickel slag fine aggregate (FS)	3.51	3.05	0.6

**Table 2:** Mix proportions of cement mortar.

Mix	NS (%)	AMF (kg/m^3^)	W/C (%)	Water (kg/m^3^)	Cement (kg/m^3^)	NS (kg/m^3^)	BS (kg/m^3^)	FS (kg/m^3^)
Control	100	0	50	170	340	784	0	0
N50F0	50	0	50	170	340	392	212	230
N50F1	50	10	50	170	340	392	212	230
N50F2	50	20	50	170	340	392	212	230
N50F3	50	30	50	170	340	392	212	230
N00F0	0	0	50	170	340	0	424	460
N00F1	0	10	50	170	340	0	424	460
N00F2	0	20	50	170	340	0	424	460
N00F3	0	30	50	170	340	0	424	460

## Data Availability

Not applicable.
